# Early childhood caries in preschool children of Kosovo - a serious public health problem

**DOI:** 10.1186/1471-2458-10-788

**Published:** 2010-12-24

**Authors:** Agim Begzati, Merita Berisha, Kastriot Meqa

**Affiliations:** 1 Department of Pedodontics and Preventive Dentistry, School of Dentistry, Medical Faculty, University of Prishtina, Prishtina, Republic of Kosovo; 2 National Institute of Public Health of Kosovo, Department of Social Medicine, Medical Faculty, University of Prishtina, Prishtina, Republic of Kosovo; 3 Department of Periodontology and Oral Medicine, School of Dentistry, Medical Faculty, University of Prishtina, Prishtina, Republic of Kosovo

## Abstract

**Background:**

Even though it has been widely studied, early childhood caries (ECC) remains a serious public health problem, especially in countries where there is no national program of oral health assessment and no genuine primary oral health care, such as in Kosovo. The purpose of this study was to assess the prevalence of ECC and analyze caries risk factors.

**Methods:**

The subjects were 1,008 preschool children, selected by stratified random cluster sampling, in the municipality of Prishtina, capital of Kosovo. Data were collected through clinical examination and interviews. Dmft data were recorded according to WHO criteria. Bacterial examination (CRT bacteria test) and plaque test of Greene-Vermillion were used.

**Results:**

The mean dmft of preschool children was found to be 5.8. The prevalence of ECC was 17.36%, with a mean dmft of 11 ± 3.6. Streptococcus mutans prevalence in ECC children was 98%. A significant correlation between dmft and S mutans counts (≥10^5 ^CFU/mL saliva) was demonstrated. A correlation was also found between daily sweets consumption and dmft in children with ECC (*P *< 0.001). Comparing the dmft of ECC children and duration of bottle feeding showed a statistical correlation (*P *< 0.001). The mean plaque test was 1.52. None of the examined children had ever used fluoride.

**Conclusion:**

The prevalence of ECC was high among preschool children in the municipality of Kosovo. We recommend increasing parents' knowledge of proper feeding habits and oral health practices, and increasing preschool children's accessibility to dental services.

## Background

Kosovo, the youngest European country, lies in the Balkan Peninsula in Southeastern Europe. The population of Kosovo in 2000 was estimated at 2 million [1], with 32.8% of the population being age 14 or younger [2]. The health care system is facing difficult organizational issues, with many problems and challenges ahead. There are no concrete activities in preventive dentistry organized by Kosovo's Ministry of Health. Some preventive activities are accomplished by the Group for Public Oral Health Promotion, established in 2000 and supported by nongovernmental organizations.

During the promotion of oral public health in urban kindergartens, the presence of extensive dental disease in children, known as early childhood caries (ECC), was recorded.

ECC is an acute, rapidly developing dental disease occurring initially in the cervical third of the maxillary incisors, destroying the crown completely. Early onset and rampant clinical progression makes ECC a serious public health problem. Due to varying clinical, etiological, localization, and course features, this pathology is found under different names such as labial caries (LC), caries of incisors, nursing bottle mouth, rampant caries (RC), nursing bottle caries (NBC), nursing caries, baby bottle tooth decay (BBTD), early childhood caries (ECC), rampant infant and early childhood dental decay, and severe early childhood caries (SECC) [3-14].

According to Davis, the definition of this pathology has always been complex and "difficult to be described, but when it is seen, you know what it's about" [15]. Up to now there have been many proposals for definition and diagnostic criteria, described in detail by Ismail & Sohn [16].

The preferred and most commonly used term today is early childhood caries (ECC), proposed by the Centers for Disease Control and Prevention (CDC) [11].

The prevalence of ECC varies in different countries, which may depend on the diagnostic criteria. While in some developed countries having advanced programs for oral health protection, the prevalence of ECC is around 5% [7,8,17-19]. In some countries of Southeastern Europe (Kosovo's neighbors), this prevalence reaches 20% (Bosnia) and 14% (Macedonia) [20,21]. Much higher ECC prevalence has been reported for such places as Quchan, Iran (59%) [22] and Alaska (66.8%) [23]. In American Indian children the prevalence is 41.8% [23]. Similarly, in North American populations, the prevalence in high-risk children ranges from 11% to 72% [24].

Numerous biological, psychosocial, and behavioral risk factors are involved in the etiology of ECC, supporting the multifactorial character of the disease [25-27]. Based on this concept, dental caries can be defined as demineralization of tooth tissue consequent to a dental infection that is dependent on frequent exposure to fermentable carbohydrates and is influenced by saliva and fluoride and other trace elements [14].

A very important role is attributed to the bacterium Streptococcus mutans-called "the window of infection" [28], in that it is responsible for the primary oral infection in the first phase of ECC [29,30].

Dietary habits are also deeply implicated in the development of ECC, despite the fact that it is considered an infectious disease [31]. Consumption of sweets with high concentrations of glucose, saccharine, or fructose, especially if taken in processed juices [32], and their prolonged intake play an important role in caries development in children with ECC [33].

The purpose of this study was to evaluate the prevalence of ECC and various caries risk factors such as quantity of cariogenic S mutans colonies, oral hygiene, sweets preference, bottle feeding in preschool children, and fluoride use.

## Methods

Included in the study were 1,008 children of both sexes, from 1 to 6 years of age, from 9 kindergartens of Prishtina, capital of Kosovo. The sample was random, representing 80% of all kindergarten children. The sample size was calculated with a confidence level of 95% and a confidence interval of 2.

### Dental examination and diagnostic criteria

The children were examined in well-lit premises, using a flashlight as the light source, and a dental mirror and dental probe, by two dentists (AB and KM). Diagnostic criteria were calibrated [34], with inter-examiner reliability resulting in kappa = 0.91, based on the examination of 35 children of different ages.

Dental caries was scored as the number of decayed, missing, or filled primary teeth (dmft).

ECC was defined as "initial occurrence of caries in cervical region of at least two maxillary incisors." Using a careful lift-the-lip examination, the presence or absence of ECC was recorded depending on the presence of "noncavity caries/white spot lesions" or "cavity caries."

In order to study the clinical and etiological aspects of ECC, a sub-sample of children with ECC was included for further analysis. The latter part of the examination, which included the clinical study of ECC development (according to ECC stages), determination of bacterial colony sampling, oral hygiene index (OHI), and filling out of the questionnaire, was conducted in the Pediatric Dentistry Clinic of the School of Dentistry.

Children with ECC were examined using the light of the dental unit, with dental mirror and probe. All examinations were carried out by AB, with intra-examiner reliability of kappa = 0.95 based on the examination of 15 children of different ages.

### Clinical course of ECC

In order to explain the clinical course of ECC, we propose the following stages in the occurrence and progression of carious lesions in ECC:

ECC_i _(initial stage)-white spot lesion or initial defect in enamel of cervix.

ECC_c _(circular stage)-lesion in the dentin and circular distribution of this lesion proximally.

ECC_d _(destructive stage)-destruction of more than half the crown without affecting the incisal edge.

ECC_r _(*radix relicta *stage)-total destruction of the crown.

The development of ECC on the maxillary incisors (at least 2) from its initial stage was monitored after a reexamination 1 year later.

### Bacterial sampling

The presence of S mutans was determined using the CRT bacteria test (Ivoclar Vivadent, Liechtenstein) on the saliva previously stimulated by chewing paraffin. Bacterial counts were recorded as colony-forming units per milliliter (CFU/mL) of saliva.

The number of bacterial colonies was graded as follows: Class 0 and Class 1 (CFU < 10^5^/mL saliva), and Class 2 and Class 3 (CFU ≥ 10^5^/mL saliva), according to the manufacturers' scoring-card (Ivoclar-Vivadent, Lichtenstein). In younger subjects, with less saliva collected, the modified spatula method was used.

The CRT bacteria test reacts more selectively than does the conventional agar-MSB method, thus allowing early SM detection [35].

### Dietary and oral hygiene questionnaire

Parents and kindergarten teachers were asked to fill out a questionnaire about the child's dietary habits, including questions regarding frequency of sweets consumption throughout the day, as well as the type of sweets. Parents answered questions about bottle feeding: first use, duration, manner, and fluid content. They were also asked if they put their children to sleep with a bottle. Regarding tooth brushing: frequency, parents' participation during brushing, how controlled, and use of fluoride-containing toothpaste and fluoride tablets.

The OHI was determined using the Plaque Test (Ivoclar Vivadent) according to the Greene-Vermilion index.

The collected data were entered in InStat 3. The level of statistical significance was set at *P *= 0.05. Statistical testing was done using the One-Way ANOVA test, and *t *test.

This study was approved by the Ethical Board of the University Dentistry Clinical Centre of Kosova (ethics approval number N-163-2010). All participants' parents gave informed consent.

## Results

### ECC prevalence

**Figure 1:**
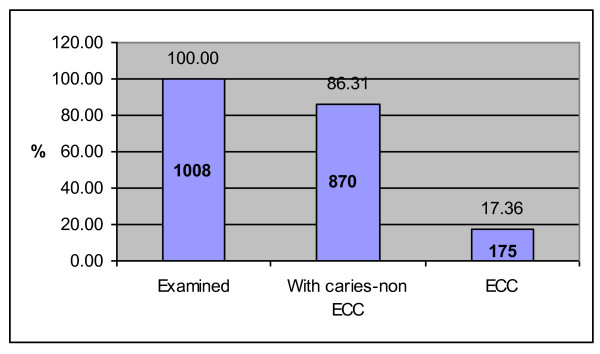
**The ECC prevalence**.

From the total 1,008 examined children aged 1-6 years, the caries prevalence expressed in terms of the caries index per person, or dmft > 0, was 86.31%, with a mean dmft of 5.8. The prevalence of ECC was 17.36%, or 175 out of 1,008 examined children (Figure 1). The sub-sample of children with diagnosed ECC consisted of 150 children out of 175 invited for further analysis. Twenty-five children of this group from different kindergartens didn't show up in the Department. The mean age of children with ECC was 3.8 ± 1.2 years. The mean dmft in children with ECC was 11 ± 3.6. There was no statistical difference of ECC prevalence between genders (*t *test = 1.81, *P *= 0.07).

As expected, the lowest mean dmft score was found at age 2 (6.47 ± 2.13), with an age-related increase in dmft of 12.8 at age 6 (Table 1).

**Table 1:** Mean dmft in children with ECC

Age	N	Mean No. Teeth Present ± SD	dmft ± SD
1-2	22	16.7 ± 1	5.5 ± 2.1
3	42	19.1 ± 1.3	9.7 ± 3.4
4	38	19.4 ± 2.1	12.8 ± 2.6
5	36	18.8 ± 3.1	12.9 ± 2.7
6	12	19.8 ± 2.4	12.8 ± 1.3
**Total**	**150**	**18.8 ± 2.2**	**11 ± 3.6**

In comparing the mean dmft in ECC children with respect to age, there was a significant statistical difference between age 2 and ages 4, 5, and 6. (One-Way ANOVA test F = 16, *P *< 0.001).

#### ECC stages

The ECC stages were not equally distributed. The most common stage present was that of radix relicta (41.7%), while the stage appearing least frequently was the initial stage (15.4%), or 27 out of 150 children with ECC. There was a significant difference between the stages of ECC (χ^2 ^= 211.1, *P *< 0.0001).

Twenty-five of the 27 children with ECC in the initial stage were reexamined 1 year after the baseline examination (2 children did not appear for reexamination due to address change). The 1-year reexamination showed that the initial stage had advanced to the circular stage in 28% of cases, destructive stage in 20%, radix relicta stage in 36%, and having been extracted due to ECC in 16% of cases (Table 2).

**Table 2:** ECC progression from initial stage at 1-year follow-up

		Baseline	Reexamination
	N	27	25

	Mean dmft ± SD	5.1 ± 1.8	8.8 ± 0.7

ECC stages	Initial stage (N, %)	(27) 100%	(0) 0%
	
	Circular stage (N, %)	/	(7) 28%
	
	Destructive stage (N, %)	/	(5) 20%
	
	Radix relicta stage (N, %)	/	(9) 36%
	
	Extraction (N, %)	/	(4) 16%

Mean age of subjects with initial stage of ECC was 2 ± 0.7.

Mean dmft on reexamination showed an increase from 5.1 to 8.8 (*P *< 0.001).

#### S mutans prevalence in ECC children

Our results show that only a small number of children (2%) with ECC exhibited the absence of S mutans (Class 0). In other words, S mutans prevalence in ECC children was 98%. The lowest class (Class 1) was recorded in 5% of the ECC children, while classes that represent higher risk for caries (Classes 2 and 3) were present in 34% and 59%, respectively, of ECC children (Table 3).

**Table 3:** S mutans distribution in ECC children

*S mutans*	N	%	Mean dmft ± SD	*T *test
***S mutans *class**				
Class 0	3	2%	3 ± 0	
Class 1	8	5%	5.4 ± 2.0	*P *< 0.001
Class 2	51	34%	9.1 ± 3.0	
Class 3	88	59%	12.8 ± 2.5	

***S mutans *values in CFU/mL saliva**				
< 10^5 ^(0 and 1)	11	7%	4.7 ± 1.1	*T *= 5.5
≥ 10^5 ^(2 and 3)	139	93%	11.5 ± 3.2	*P *< 0.001

Total	150	100%	**11 ± 3.6**	

Only 11 children with ECC exhibited a low level of S mutans colonies (CFU < 10^5^), with the mean dmft of this group being 4.7. The groups with higher CFU of S mutans (Classes 2 and 3), representing 93% of the children, had a mean dmft of 11.5 ± 3 (Table 3). Comparing the mean dmft of ECC children by S mutans classes of CFU showed a significant difference between Class 1 and Classes 2 and 3 (t = 5.5, *P *< 0.001).

### Sweets consumption

We found that the frequency of sweets consumption in approximately 93% of the children was 1-3 or more times a day. Sweets consumption between meals and during kindergarten hours was common.

There was a statistical correlation between daily sweets consumption and dmft in children with ECC (F = 7.26, *P *< 0.001) (Table 4).

**Table 4:** Sweets consumption, bottle feeding, and oral hygiene in ECC children

	N	%	Mean dmft ± SD	One -way ANOVA test
**Sweets consumption**				
Not every day	10	7%	6.3 ± 2.9	
Once a day	23	15%	9.9 ± 3.8	F = 7.26
Twice a day	60	40%	11.1 ± 0	*P *< 0.001
Three and more/day	57	38%	12.3 ± 2.9	

**Bottle feeding**				
No	9	6%	4.3 ± 1.8	
Les than 1 year of age	10	7%	6.5 ± 2.1	F = 20.83
1-2 years of age	72	48%	11.8 ± 3.2	*P *< 0.001
3+ years of age	59	39%	11.9 ± 2.6	

**Tooth brushing frequency**				
0	78	52%	11.1 ± 3.7	F = 2.10
Once a day	59	39%	10.8 ± 3.4	*P *= 0.106
Twice a day	13	9%	11.3 ± 3.7	

**OHI**				
1	81	54%	10.5 ± 3.8	F = 1.84
2	60	40%	11.3 ± 3.2	*P *= 0.165
3	9	6%	13.2 ± 3.6	

Total	150	100%	**11 ± 3.6**	

#### Bottle feeding in ECC children

Most of the children with ECC represent subjects who are bottle fed up to age 2 (48%) and 3 and up (39%). Of the children with ECC, 6% were not bottle fed and 7% were bottle fed up to age 1. Comparing the dmft of ECC children with regard to duration of bottle feeding shows statistical correlation (F = 20.83, *P *< 0.001) (Table 4).

#### Tooth brushing and the OHI in ECC children

Regarding the frequency of tooth brushing, around 52% of the children did not brush at all, but there was no statistical difference in dmft in terms of frequency of brushing (F = 2.10, *P *= 0.106).

The mean plaque test was 1.52. No child recorded OHI-1. Although a dmft of 13 was found in children with OHI-3, no significant difference was found when comparing the dmft with respect to OHI (F = 2.52, *P *= 0.085) (Table 4).

### Fluoride use

None of the mothers reported giving fluoride to their children.

## Discussion

The oral health of Kosovar children is in a deplorable state, exhibiting high caries prevalence in general and high prevalence of ECC in particular.

According to WHO criteria [36], the mean dmft in the preschool children in general and the mean dmft in the group with ECC (5.8 and 11, respectively), may be considered very high. Furthermore, the unfavorable status of dental health services, caries risk factors, and clinical consequences, make ECC in Kosovar children a serious public health problem.

### Risk factors of ECC

As the data from the literature show, the role of S mutans in the etiology of ECC, especially in the initial phase, is very crucial [28,29]. These data also demonstrate the high prevalence of this bacterium in preschool children. S mutans is found at the earliest ages, with the prevalence of 53% in 6- to 12-month-old children [37], 60% in 15-month-olds [38], 67% in 18-month-old Swedes [39], and 94.7% in 3- to 4-year-old Chinese [40]. Almost all preschool urban Icelandic children were found to carry S mutans [41]. According to the studies of Ge and Caufield, all S-ECC children were S mutans-positive [42]. Borutta [43], found that in 80% of children (3 years old) diagnosed with caries, the presence of S mutans was demonstrated, while higher counts of this bacterium were found in children with ECC.

The high prevalence of S mutans was also demonstrated in our study: 98% of preschool children. Expressed in colony-forming units (CFU/mL saliva), 93% of the ECC children in our study had a high S. mutans counts (CFU > 10^5^). Higher salivary counts of *S. mutans *have been correlated with high dmft values (11.5) in our study. This significant correlation between high dmft or caries experience and high S mutans counts has been demonstrated in other studies [44-47].

In our study, the sweets consumption of children with ECC was very high. Almost 4/5 of ECC children have sweet snacks more than twice a day. It is of great concern that kindergartens as educational institutions do not have a more serious approach to a healthy diet and reduction of sugary food. On the contrary, at least once a day, sweet food (jam, chocolate, cream, biscuits, or cake) is served to children. Also, serving of this food is very common between meals. The literature also shows a high consumption of sweets between meals [48] and high caries values in children who have frequent sweets [49].

Another important factor in the etiology of ECC is bottle feeding, which is accompanied by high salivary counts of *S mutans*. The relationship between bottle usage and salivary counts of S mutans [50] has been reported. In our children, the duration of bottle feeding with sweetened milk or juice is very long, wherein nearly 4/5 of children are bottle fed from 1 to 3 and more years.

Another harmful practice is putting children to sleep with a juice-filled bottle, which is practiced in 2/3 of children with ECC, although Johnsen has reported that 78% of parents of children with ECC had attempted to substitute water for a cariogenic liquid (e.g., apple juice, formula) in the bedtime nursing bottle [51]. A review of the literature from the etiological point of view of ECC shows that "the use of a bottle at night" is not the only cause of ECC [52].

Oral hygiene habits established at the age of 1 can be maintained throughout early childhood [53]. There is a high level of negligence in the oral hygiene of our children. More than half do not brush their teeth at all, exhibiting a very high OHI (1.52). The importance of the primary dentition of oral health promotion must be focused on the education of mothers to motivate their children for oral hygiene. Unfortunately, we found "bad conviction" of mothers regarding primary teeth that they will be replaced, thus neglecting the care for children's teeth. Data from the literature show that cooperation of mothers is very important in overcoming the belief that the deciduous dentition can be neglected [54].

From the answers of mothers concerning fluoride use, we ascertained a marked lack of knowledge about the benefits of this agent in maintaining healthy tooth structure. This information gap can be inferred from their answers. When asked, "Do you give fluoride tablets to your child?" their answers were stated as if they have been asked about some medication: "I give those tablets to my child as needed." The absence of fluoride in Kosovo's municipal drinking water may highly influence caries prevalence rates in children.

Nutritional counseling, fluoride therapy, and oral hygiene may be required to prevent development of carious lesions in children. In the case of high-risk patients such as ECC children with a predominance of high salivary counts of S mutans, the use of either the antibacterial rinse chlorhexidine gluconate or the oral health care gel chlorhexidine has been suggested [55].

The oral health promotion and preventive measures are also influenced by social and economical factors. Statistical data from our country such as: large families (with average size of 6.5 members) [2], high unemployment rate (in 2008 it marked 45.4%, for female 56.4%), high birth rate (16%) and the lowest economical growth in the region [56], represent some of the aggravating factors when dealing with the health issues of the population, including oral health issues.

Given the complexity of factors associated with ECC, it is unfortunate that most of the interest has only been from dental organizations. The critical change needed to accomplish the necessary research into the prevention of ECC is to expand our network to include other health professionals, community leaders, national organizations serving children, and political leaders [57].

### Consequences of ECC

Scientific research suggests that the development of ECC occurs in 3 stages. The first stage is characterized by a primary infection of the oral cavity with ECC. The second stage is the proliferation of these organisms to pathogenic levels as a consequence of frequent and prolonged exposure to cariogenic substrates. Finally, a rapid demineralization and cavitation of the enamel occurs, resulting in rampant dental caries [24].

A 1-year follow-up of ECC development from the initial stage, representing decay at the enamel level and its progression to more destructive stages, shows even development in all affected teeth. It is quite an acute development, because in 2/3 of the children, the ECC has progressed to more complicated stages (destructive and radix relicta stages). Within 1 year, the dmft values have increased to 3.7. Consecutively, these children commonly experience pain from pulpitis, gangrene, and apical periodontitis. Also, these conditions are often followed by abscesses and cellulitis, sometimes with phlegmona, seriously endangering the child's general health. De Grauwe, in describing the progression of ECC, has noticed that the development of caries from the enamel to the dentin level can occur within 6 months [58].

The rapid development of ECC and its clinical appearance, especially in primary incisors, identifies it in its initial stages as a risk factor for future caries in the primary and permanent dentitions [59].

Children with congenital heart anomalies are frequent patients in our departments, some of them exhibiting severe ECC.

There is strong evidence that untreated dental disease is an important etiological factor in the pathogenesis of infective endocarditis, a condition that still carries a high risk of mortality [60].

## Conclusions

Caries in general and ECC in particular represent a serious public health problem for Kosovar children. Kosovar society is lacking an efficient health care system, and it has no preventive program organized by governmental institutions.

Some volunteer initiatives, such as activities of the Group for Public Oral Health Promotion, are not sufficient for achieving optimal outcome, so it is important to introduce oral health promotion.

Primary prevention must start in the prenatal stage to fulfill the needs of pregnancy. Parents should be encouraged to avoid bad feeding habits and to instruct and supervise their children in tooth brushing. Mothers should be instructed to use the lift-the-lip technique to spot the white-spot lesions as first signs of dental caries. Newly erupted teeth must be treated with fluoride agents, and, as needed, antimicrobial agents containing chlorhexidine and thymol. Further investigation is needed to assess the effectiveness of new intervention strategies beyond traditional measures that are not strictly dependent on access to dental professional providers.

## Competing interests

This was not an industry-supported study. The authors declare that they have no competing interests.

## Authors' contributions

AB contributed substantially to the conception and design of the project, carried out the dental examinations, and participated in the questionnaire collection. He collected and reviewed the literature and consulted professionals in the field of study from other countries. He prepared the text. KM participated in the dental examination and in questionnaire collection, was involved in drafting and revising the manuscript, and translated it into English. MB consulted on public health issues, statistical analysis, and statistical comments of the manuscript. All authors have read and approved the final manuscript

## Pre-publication history

The pre-publication history for this paper can be accessed here:

http://www.biomedcentral.com/1471-2458/10/788/prepub
